# A neural marker of the human face identity familiarity effect

**DOI:** 10.1038/s41598-023-40852-9

**Published:** 2023-09-28

**Authors:** Xiaoqian Yan, Angélique Volfart, Bruno Rossion

**Affiliations:** 1 Institute of Science and Technology for Brain-Inspired Intelligence, Fudan University, Shanghai, 200433 China; 2 Université de Lorraine, CNRS, 54000 Nancy, France; 3 Psychological Sciences Research Institute, Université Catholique de Louvain, 1348 Louvain-la-Neuve, Belgium; 4 Faculty of Health, School of Psychology and Counselling, Queensland University of Technology, Brisbane, QLD 4059 Australia; 5 Université de Lorraine, CHRU-Nancy, Service de Neurologie, 54000 Nancy, France

**Keywords:** Perception, Human behaviour

## Abstract

Human adults associate different views of an identity much better for familiar than for unfamiliar faces. However, a robust and consistent neural index of this behavioral face identity familiarity effect (FIFE)—not found in non-human primate species—is lacking. Here we provide such a neural FIFE index, measured implicitly and with one fixation per face. Fourteen participants viewed 70 s stimulation sequences of a large set (n = 40) of widely variable natural images of a face identity at a rate of 6 images/second (6 Hz). Different face identities appeared every 5th image (1.2 Hz). In a sequence, face images were either familiar (i.e., famous) or unfamiliar, participants performing a non-periodic task unrelated to face recognition. The face identity recognition response identified at 1.2 Hz over occipital-temporal regions in the frequency-domain electroencephalogram was 3.4 times larger for familiar than unfamiliar faces. The neural response to familiar faces—which emerged at about 180 ms following face onset—was significant in each individual but a case of prosopdysgnosia. Besides potential clinical and forensic applications to implicitly measure one’s knowledge of a face identity, these findings open new perspectives to clarify the neurofunctional source of the FIFE and understand the nature of human face identity recognition.

## Introduction

The ability to identify people by their face—Face Identity Recognition (FIR)—is an important social skill for human beings. Yet, FIR is extremely challenging for the human brain. One key reason for this challenge is that the appearance of a given face identity can vary substantially across viewing conditions^1–3^. Beyond the ability to discriminate different face identities, FIR therefore strongly relies on the ability to *generalize* across highly variable views of the same face identity.

Over the last three decades, many behavioral studies have shown that generalization across different views (i.e., matching different pictures) of the same face identity is much better for face identities that have been encoded in memory (i.e., familiar faces) than for unfamiliar faces^1,4–7^. This face identity familiarity effect (FIFE)—which is not found in non-human primate species^8,9^ and may therefore be specific to humans^10,11^—is typically described in behavioral studies in which participants are required to explicitly report whether two or more face images presented concurrently are from the same identity or not^4,7^. The explicit tasks used in these studies are performed with little or no time-constraint and, in addition to differential eye movement explorations between familiar and unfamiliar faces, may therefore involve many different cognitive processes (visual perception, attention, semantic associations, verbalization, decisional processes, etc.). Beyond differences in face identity knowledge, this may explain why the FIFE can vary greatly across individuals as well as task conditions and instructions^3,7,12,13^. Most importantly, the source of this robust effect is not well understood and debated^14–18^.

**Figure 1:**
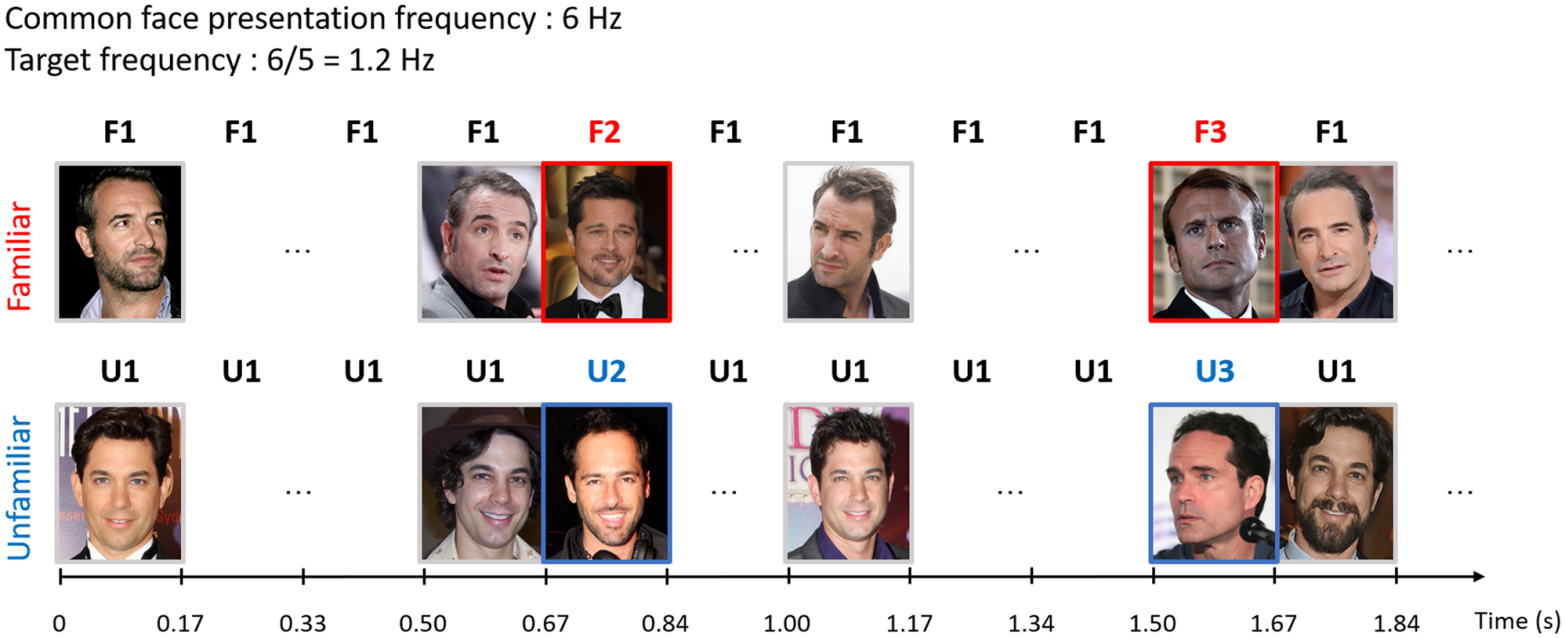
Schematic illustration of the experimental design. Different images of a particular face identity (FID1 in the familiar condition and UID1 in the unfamiliar condition, here showing a well-known French/Australian celebrity) are presented through square-wave contrast modulation with a 50% duty cycle at a fixed rate of 6 Hz (i.e., 6 images by second), with different face identities (FID2, FID3, … in the top row or UID2, UID3, … in the bottom row) embedded every 5th image (i.e., 1.2 Hz). The three celebrity faces shown in the figure for the familiar face condition are **Jean Dujardin** (Pictures licensed under the CC BY-SA 3.0. Attribution: Georges Biard, and Avatoru), **Brad Pitt** (Picture licensed under the CC BY-SA 4.0. Attribution: Toglenn), and **Emmanuel Macron** (Picture licensed under the CC BY-SA 2.0. Attribution: European People’s Party). The three celebrity faces shown for the unfamiliar face condition in the figure are **Adam Garcia** (Pictures licensed under the CC BY-SA 2.0., CC BY-SA 2.0., CC BY-SA 4.0., and CC BY-NC-ND 2.0. Attribution: Eva Rinaldi, Eva Rinaldi, Laura Gold, and Sean James Cameron), **Alex Dimitriades** (Picture licensed under the CC BY-SA 2.0. Attribution: Eva Rinaldi), and **Jason Patric** (Picture licensed under the CC BY-SA 2.0. Attribution: Gage Skidmore). Here images shown in the figure may not be used in the experiment due to copyright limitations.

**Figure 2:**
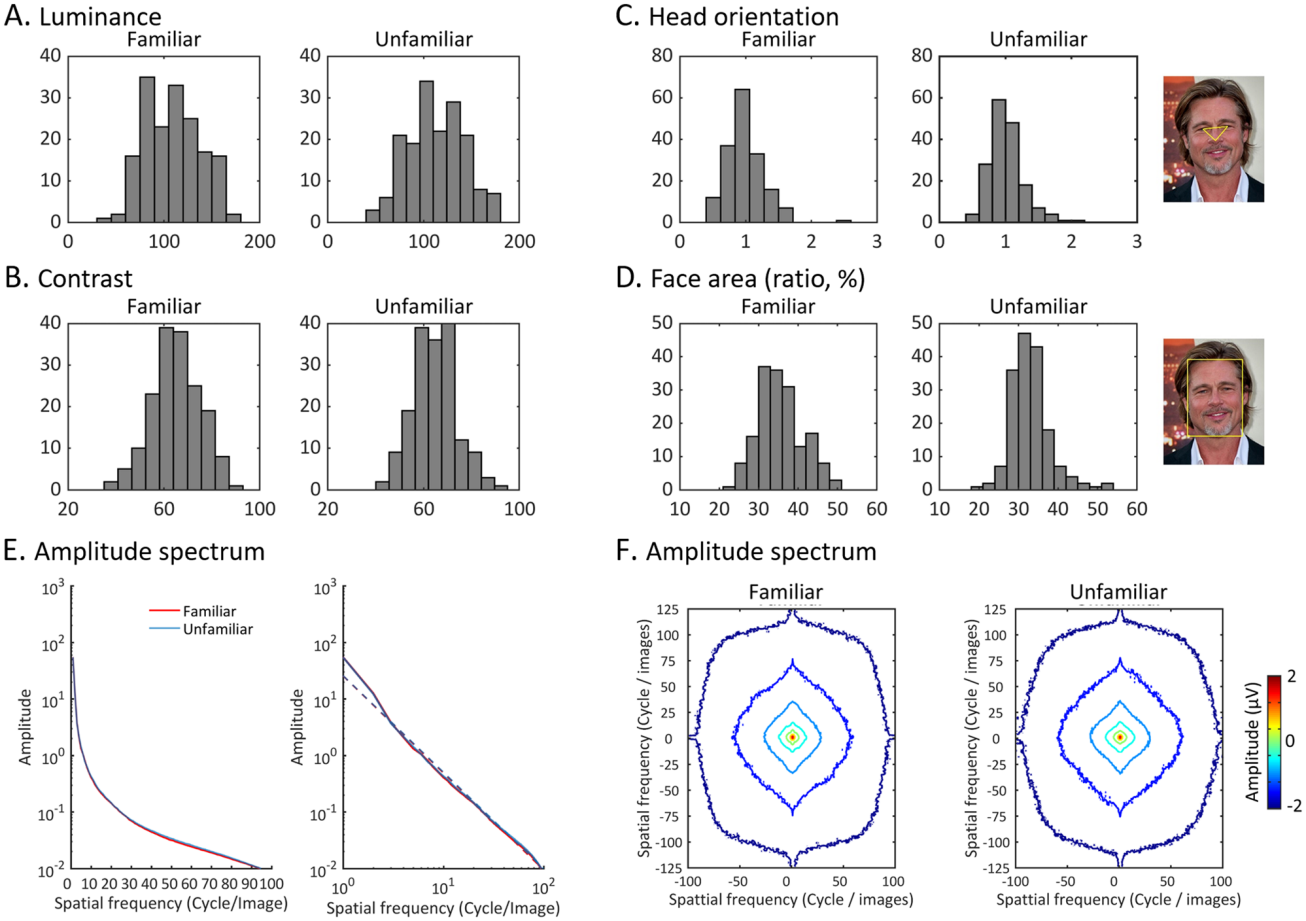
Image analyses. (**A**) Histograms showing the distribution of luminance values across face images of each stimulus set (familiar and unfamiliar faces). No significant differences in luminance were observed (p > .1) (see methods section). (**B**) Histograms showing the distribution of contrast values across face images of each stimulus set (familiar and unfamiliar faces). No significant differences in contrast were observed (ps > .1) (see methods section). (**C**) Histograms showing the head orientation distributions of the face images in each set. The head orientation of each face image was calculated with the ratio between the distance from the tip of nose to the left pupil and right pupil, respectively. A value of 1 on the x-axis indicates a frontal-view face image. A value smaller than 1 indicates a face image orientating leftward. An example measurement of nose-eye distance is shown on the right, the face identity is Brad Pitt (Picture licensed under the Creative Commons Attribution-Shared Alike 4.0 International. Attribution: Toglenn.). (**D**) Histograms showing the distribution of percentage face area (%) in the two sets. An example definition of face area is shown on the right. (**E**) Left panel: Radial averages of the amplitude spectrum of the two sets of face images. The y-axis is in log scale. The plot shows no amplitude difference across spatial frequencies between familiar and unfamiliar faces. Right panel**:** The radial averages of amplitude spectra shown in log–log scale and fitted with linear functions with the spectrum amplitudes transformed into a log–log scale (dashed lines). (**F**) Contour plots showing the mean spectral signature of faces in the two face sets.

Here we aim at shedding light on this issue by providing a quantified neural index of the FIFE, that is the difference of FIR response to familiar and unfamiliar faces, at both group and individual levels, measured implicitly and under severe time constraints (i.e., one fixation per face identity). To do so, we measured FIR with the Fast Periodic Visual Stimulation (FPVS) approach, presenting a particularly large number (n = 40) of variable natural images of the same face identity (ID1) at a fixed rate of 6 Hz, allowing one fixation per face presentation and asking participants to perform a simple orthogonal task. During this visual stimulation sequence of 70 s, natural images of other face identities (ID2, ID3, etc.) were embedded periodically every 5th image (i.e., at 6 Hz / 5 = 1.2 Hz, Fig. 1). The rationale was that if widely variable views of the same face identity are processed as a single identity, interruption by a different identity every 5th stimuli should lead to an ‘oddball’ response exactly at 1.2 Hz, quantifiable in the recorded electroencephalographic (EEG) spectrum as the summed voltage amplitude at this pre-determined frequency and its harmonics (2.4 Hz, 3.6 Hz, etc.)^19,20^. The quantified FIR response reflects the contrast between the *generalization* of different images of the same identity and the *discrimination* of the appearance of different face identities at every 5th stimuli in each face condition. In this respect, the paradigm is similar to another FPVS paradigm using homogenized pictures of full-front unfamiliar faces of the same face identity varying slightly in image size disrupted by other unfamiliar faces of different identities, and validated in many studies in recent years^21^ (reviewed in^22^). However, critically, and in line with recent FPVS-EEG studies with familiar faces^23–26^, widely different natural images of the same face identity are presented here throughout a stimulation sequence (Fig. 1), emphasizing the generalization function of FIR compared to the discrimination function. Moreover, and importantly, participants viewed stimulation sequences with only familiar (i.e., famous) faces or only unfamiliar faces, with both types of faces being widely variable in views and yet carefully matched for physical properties (see methods and Fig. 2).

To be clear, here our goal is not to directly compare neural responses to familiar and unfamiliar faces in humans, something that has been done in several FPVS-EEG studies^23–27^ and many studies with indirect or direct neural measures including EEG (or magnetoencephalography, MEG), with mixed and various outcomes^25,28–36^. Instead, we aim at measuring the effect of familiarity on the amplitude of the “disruptive” response elicited by the appearance of the different-identity face on the generalization response elicited to different views of the same-identity face.

Given that the base rate stimulation at 6 Hz reflects many processes, we do not expect much if any difference in neural signals between familiar and unfamiliar face sequences in the present study. However, in line with behavioral evidence, we hypothesize to find a much larger FIR neural response for familiar as compared to unfamiliar faces, i.e., a neural FIFE, in every individual tested. Such findings would have both theoretical (i.e., understanding of the nature of the FIFE, and human FIR more generally) and practical (i.e., development of implicit measures of one’s knowledge of a familiar face identity) implications.

## Results

In this section, we first present EEG results in the frequency-domain in a group of 14 neurotypical participants before contrasting them with the EEG results observed in an age-matched participant (JG) whose subjective report and performance at face recognition tasks (see methods) suggest he suffers from prosopdysgnosia. We then present the time-domain EEG results observed in the group of 14 neurotypical participants.

### FIR responses (*n*1.2 Hz)

**Figure 3:**
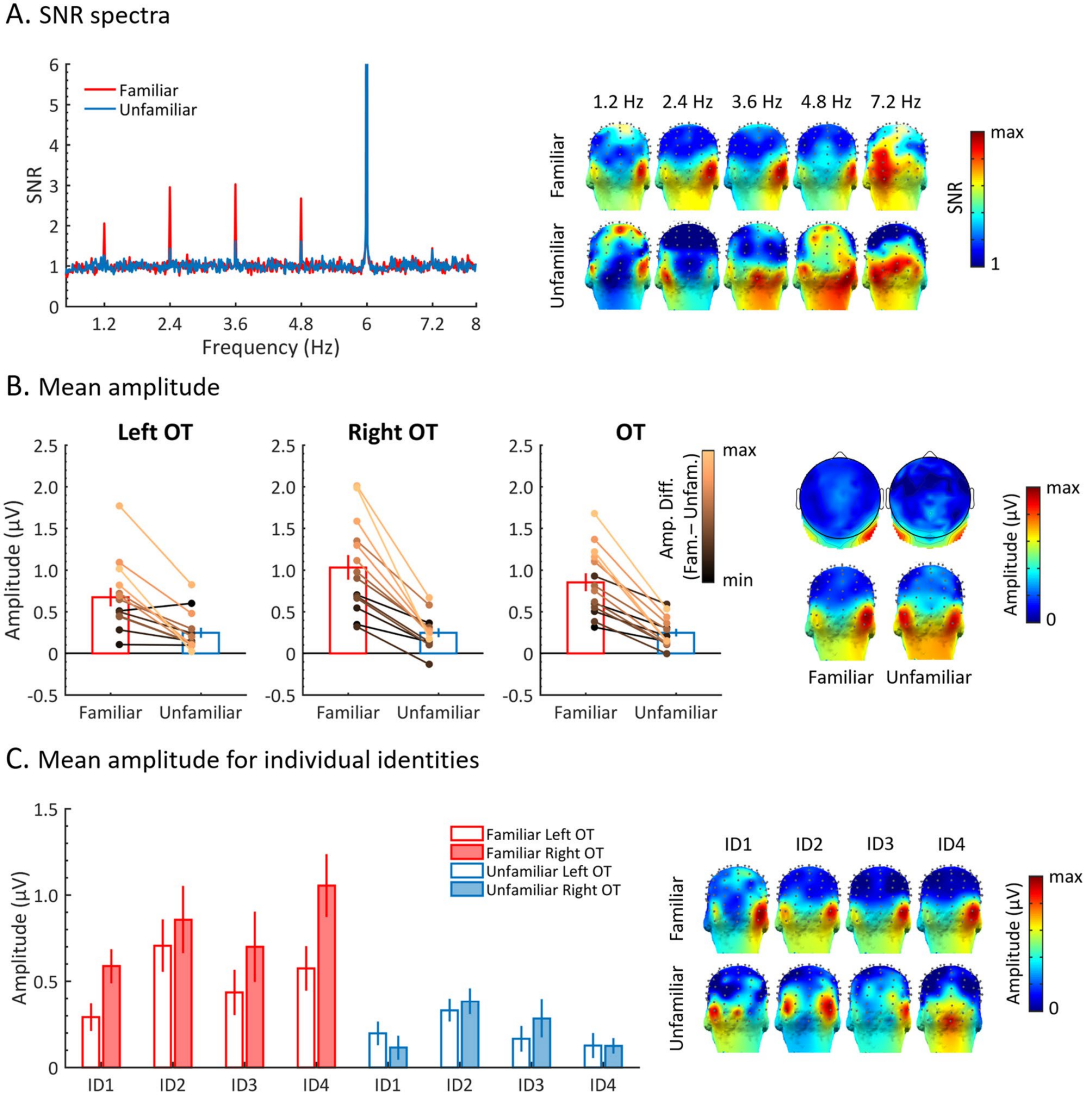
Amplitudes of FIR responses to familiar and unfamiliar faces at the group level. (**A**) SNR spectrum of the two face conditions (x-axis range: 0.5–8 Hz) over bilateral OT ROIs. Significant responses were observed at 1.2 Hz and 4 following harmonics, with the first four showing much higher SNRs to familiar versus unfamiliar faces. The response SNR at 6 Hz in both face conditions were higher than 20 but the y-axis is chunked here to better display the FIR response ranges of the responses at 1.2 Hz and harmonics. Three-D scalp topography maps (posterior view) for each significant harmonic are shown on the right. The color scale is adjusted to the maximum SNR for each map. (**B**) Mean amplitudes with standard errors for both face conditions over left, right, or both OT ROIs. The FIR responses for each participant at both conditions are shown with circle markers and linked with a line whose color is scaled by the amplitude difference between the two face conditions across participants. Two-D scalp topography maps for summed-harmonic response are shown on the right. Three-D scalp topography maps for summed-harmonic response are shown on the right below the two-D maps. (**C**) FIR responses are displayed for each individual base face identity (4 familiar, 4 unfamiliar) used in the familiar or unfamiliar face condition over the left and right OT ROIs. Larger response amplitudes are observed for familiar face identities as compared to unfamiliar face identities. White bars represent FIR responses over the left OT ROI and red (blue) filled bars represent FIR responses over the right OT ROI. Three-D topographies for each condition and identity are shown on the right.

Clear FIR responses in both face conditions were found at the group level, especially over bilateral occipito-temporal (OT) regions (Fig. 3A), but the SNR at 1.2 Hz and 4 following harmonics (i.e., 2.4, 3.6, 4.8, and 7.2 Hz) in the familiar face condition was much higher than in the unfamiliar face condition. For both conditions, there was only a small response at the 6th harmonic (7.2 Hz), with no obvious difference between conditions (Fig. 3A).

The FIR responses (baseline-corrected amplitudes in microvolts) to the two face conditions were analyzed over all 128 channels as well as bilateral OT ROIs. Across all 128 channels, the FIR responses to both face conditions (familiar, *M* = 0.28 ± 0.13; unfamiliar, *M* = 0.07 ± 0.06) were significantly above zero (familiar, *t*_(13)_ = 8.36, *p* < 0.001; unfamiliar, *t*_(13)_ = 4.19, *p* < 0.001), but the FIR response to familiar faces was 4 times larger than the response to unfamiliar faces, *t*_(13)_ = 6.96, *p* < 0.001. Over the OT regions, a repeated measures ANOVA with *Hemisphere* (left, right) and *Familiarity* (familiar, unfamiliar) as within-subjects factors (Fig. 3B) showed a significantly larger FIR response to familiar faces, *F*(1,13) = 50.35, *p* < 0.001, η^2^ = 0.8 (familiar, *M* = 0.86 ± 0.4; unfamiliar, *M* = 0.25 ± 0.18). Although the FIR response to unfamiliar faces was significantly above zero over bilateral OT regions (left: *t*_(13)_ = 4.04, *p* < 0.001; right: *t*_(13)_ = 4.68, *p* < 0.001), it was only about 29% of the response to familiar faces (i.e., a 3.44 times larger response for familiar as compared to unfamiliar faces). In addition, there was a significant interaction of *Hemisphere* × *Familiarity, F*(1,13) = 8.49, *p* < 0.05, η^2^ = 0.4. Decomposing the interaction revealed a right hemisphere dominance of the response in the familiar face condition (*t*_(13)_ = 2.42, *p* < 0.05, bonferroni-corrected), but not in the unfamiliar face condition (*t*_(13)_ = 0.03, *p* > 0.1, bonferroni-corrected).

A separate repeated measures ANOVA with *Identity* (8 levels) and *Hemisphere* (left, right) as within-subjects factors (Fig. 3C) showed a significant main effect of *Identity, F*(7,91) = 9.99, *p* < 0.001, η^2^ = 0.435, and a significant interaction of *Identity* × *Hemisphere, F*(7,91) = 2.42, *p* < 0.05, η^2^ = 0.16. Further analysis showed that for the familiar face identities FID1 (i.e., *J. Dujardin*,) and FID4 (i.e., *N. Aliagas*), the amplitude in the right hemisphere was significantly larger than in the left hemisphere (FID1, *t*_(13)_ = 2.24, *p* < 0.05, uncorrected, FID4, *t*_(13)_ = 2.89, *p* < 0.05, uncorrected).

**Figure 4:**
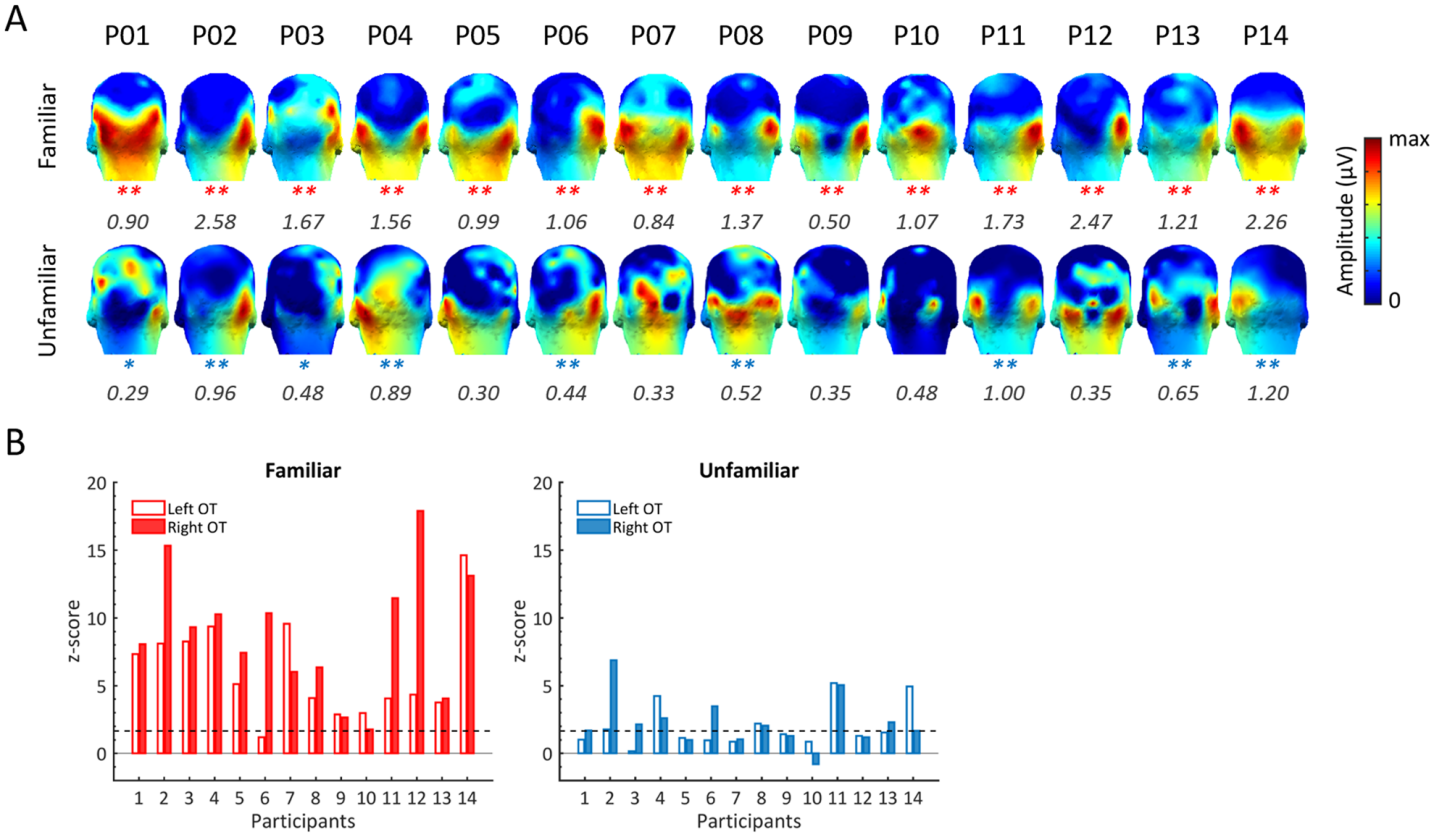
FIR responses at the individual participant level over the occipito-temporal regions. (**A**) Three-D topographies displaying the baseline-corrected amplitude of the FIR response for both face conditions. Asterisks below the scalp topographies indicate a significant FIR response (*z-score > 1.64, p < .05, one-tailed; **z-score > 2.32, p < .01, one-tailed). The color scale is adjusted to show the maximum amplitude for each topography. The maximum amplitude for each topography map is shown below each map. (**B**) Z-scores of the individual FIR response in each OT ROI. The dashed line indicates the z-score threshold at 1.64 (p < .05).

Apart from the difference in hemispheric lateralization, the group-level scalp topographies were very similar between the two conditions (see below), with a peak over OT regions. Yet, there were variations across individuals (Fig. 4).

Impressively, at the individual participant level, significant FIR responses to familiar faces were found in 100% of participants over both OT ROIs (except for P06, for which the effect was not significant in the left hemisphere, Fig. 4). In contrast, only 5 of the same 14 participants showed significant FIR responses to unfamiliar faces over both OT regions, with 9 participants showing significant responses in the right hemisphere and 5 in the left hemisphere. All participants showed a numerically larger response to familiar as compared to unfamiliar faces (range: 1.6 to 73 times the response amplitude). 86% of participants (12 out of 14) showed a statistically significantly larger FIR response to familiar versus unfamiliar faces over the right OT region, and 71% over the left OT region. The two participants who did not show a significantly larger response to familiar than unfamiliar faces on the group-defined ROIs (P9 and P13) nevertheless presented with a numerical difference between the two conditions (their FIR response to familiar faces being 2 times and 1.6 times greater than to unfamiliar faces, respectively). Compared to other participants, these two individuals presented with the most focal OT responses, with similar topographies for familiar and unfamiliar faces (Fig. 4).

In general, participants who showed a larger FIR response to familiar faces over the OT regions also showed a larger response to unfamiliar faces, as indicated by a significant correlation analysis across individuals, *r* = 0.62, *p* < 0.05.

For the sake of completeness, we also ran correlation analyses between the magnitude of the FIR oddball responses in the OT ROI (averaged across 10 channels) in the familiar face condition and the level of familiarity with the base or oddball faces that were presented to the participants (as assessed by familiarity ratings, see methods), the mean familiarity with all faces or the difference in familiarity between base and oddball faces. Even at liberal thresholds of uncorrected *p* < 0.05, there were no significant correlations between the neural response and familiarity ratings to base faces, *r* = −0.29, *p* = 0.32, familiarity ratings to oddball faces, *r* = 0.29, *p* = 0.31, mean familiarity ratings to oddball and base faces, *r* = 0.18, *p* = 0.53, or difference in familiarity ratings between base and oddball faces, *r* = −0.46, *p* = 0.1. There was no significant correlation either between the difference in familiarity ratings of oddball and base faces and the magnitude of the EEG difference between familiar and unfamiliar face responses, *r* = −0.42 (negative trend), *p* = 0.13, or between mean ratings for oddball and base faces and the magnitude of EEG difference, *r* = 0.09, *p* = 0.75. In addition, there was no significant correlations between the amplitude of the FIR responses in the familiar face condition and the BFRT-c scores, r = 0.21, r = 0.48, or the BFRT-c response times, *r* = −0.1, *p* = 0.75. For the unfamiliar face condition, the FIR responses in the OT ROIs were not significantly correlated with the BFRT-c results either (scores, *r* = 0.23, *p* = 0.43; RTs, *r* = 0.27, *p* =  = 0.35).

### General visual responses (*n*6 Hz)

**Figure 5:**
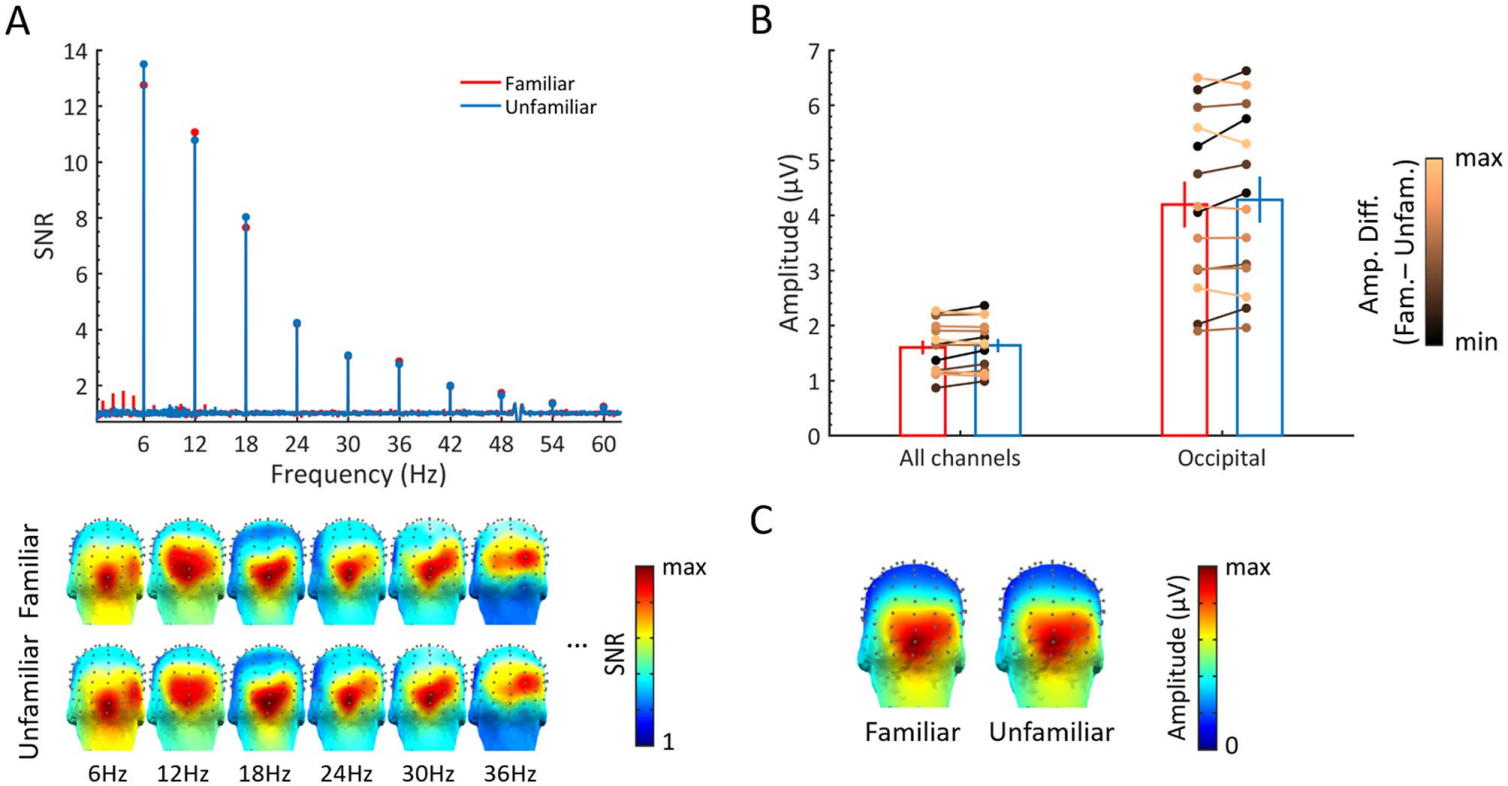
Grand-averaged general face presentation response at 6 Hz and 9 following harmonics (up to 60 Hz) over the middle occipital ROI region. (**A**) SNR spectra of the two face conditions. Three-D scalp topography maps of the first 6 significant harmonics are shown below the spectrum plot for each face condition. (**B**) Mean of baseline-corrected amplitudes for both face conditions over the middle occipital ROI, and across all channels. Error bars indicate standard errors of the mean. Individual response amplitudes for each condition are indicated with circle markers and linked with a line whose color is scaled by the amplitude difference between the two face conditions. (**C**) Three-D scalp topography maps for summed-harmonic responses in both face conditions.

Significant general face presentation responses were observed at 6 Hz and its following nine harmonics (i.e., up to 60 Hz) for both face conditions (Fig. 5). A repeated measures ANOVA with *ROIs* (Occipital, all channels) and *Familiarity* (familiar, unfamiliar) as within-subjects factors showed a larger response over the middle occipital region than across all 128 channels, *F*(1,13) = 62.7, *p* < 0.001, η^2^ = 0.83. However, there was no response difference between the two face conditions, *F*(1,13) = 2.55, *p* > 0.1, η^2^ = 0.16, i.e., no absolute response difference between familiar and unfamiliar faces.

### EEG results for participant JG

**Figure 6:**
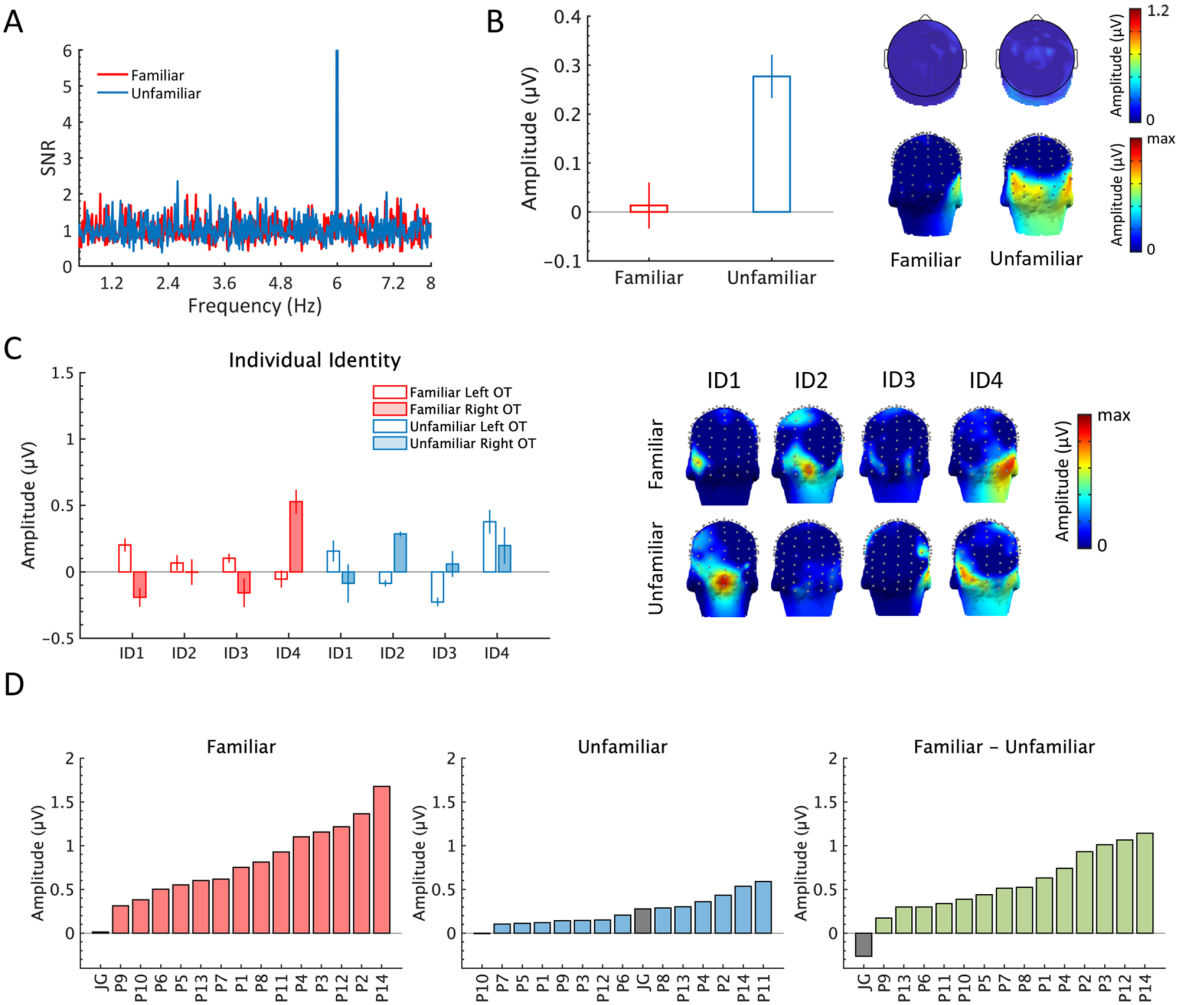
FIR responses to familiar and unfamiliar faces in participant JG. (**A**) SNR spectrum of the two face conditions (x-axis range: 0.5–8 Hz) over bilateral OT ROIs. A small significant response was observed at 3.6 Hz only in the unfamiliar face condition. The response SNR at 6 Hz in both face conditions was higher than 20 but the y-axis is chunked here to better display the FIR response ranges of the responses at 1.2 Hz and harmonics. (**B**) Mean amplitudes with standard errors across 10 OT channels for both face conditions. Three-D scalp topography maps (posterior view) for both face conditions are shown on the right. The color scale is adjusted to the maximum amplitude for each map. (**C**) JG’s FIR responses are displayed for each individual base face identity used in the familiar and unfamiliar face condition. Three-D scalp topography maps (posterior view) of FIR responses for each base identity in both face conditions are shown on the right. The color scale is adjusted to the maximum amplitude for each map. (**D**) The distribution of FIR response amplitudes is presented for all participants including JG for the familiar (left panel) and unfamiliar (middle panel) face conditions. The distribution of response amplitudes is also shown for the difference between the familiar and unfamiliar conditions (right panel).

Across all 128 channels, the summed-harmonics responses of participant JG in both face conditions were not significantly above noise (familiar face condition, z-score = −0.86, amplitude = −0.04 ± 0.09 µV; unfamiliar face condition, z-score = 0.65, amplitude = 0.03 ± 0.14 µV). Over the OT ROI defined in neurotypical participants, JG's FIR response to unfamiliar faces was significantly above noise (z-score = 3.79, amplitude = 0.28 ± 0.14 µV) due to a small significant response at 3.6 Hz in the unfamiliar face condition (Fig. 6A,B). However, there was no response to familiar faces (z-score = 0.16, amplitude = 0.01 ± 0.15 µV). JG’s response to individual face identities (used as base image in each condition) showed relatively larger responses to only one familiar face identity (i.e., FID4, N. Aliagas; *M* = 0.24 µV) and one unfamiliar face identity (i.e., UID4, T. Neuvic; *M* = 0.29 µV) (Fig. 6C). JG’s response amplitudes as compared to typical participants’ are displayed in Fig. 6D. Immediately after the EEG recording, participant JG completed the same face familiarity questionnaire as the typical participants (see methods). Among all 14 familiar face identities, JG identified three (N. Aliagas, L. DiCaprio, and E. Macron), which was in strong contrast with the typical participants whose mean number of recognized face identities was 10.3. JG did not recognize any of the unfamiliar face identities, which was expected. For the three correctly recognized familiar faces, JG gave a mean familiarity rating of 7/10 (± 1).

In summary, we demonstrated here that this face individuation FPVS paradigm is sensitive to face recognition abilities. Participant JG showed significantly reduced FIR responses compared to the typical participants. More importantly, these findings provide further evidence that the larger response amplitudes to the familiar versus unfamiliar faces in neurotypical participants were unlikely biased by low-level image characteristics.

### Spatio–temporal dynamics of the FIR responses

**Figure 7:**
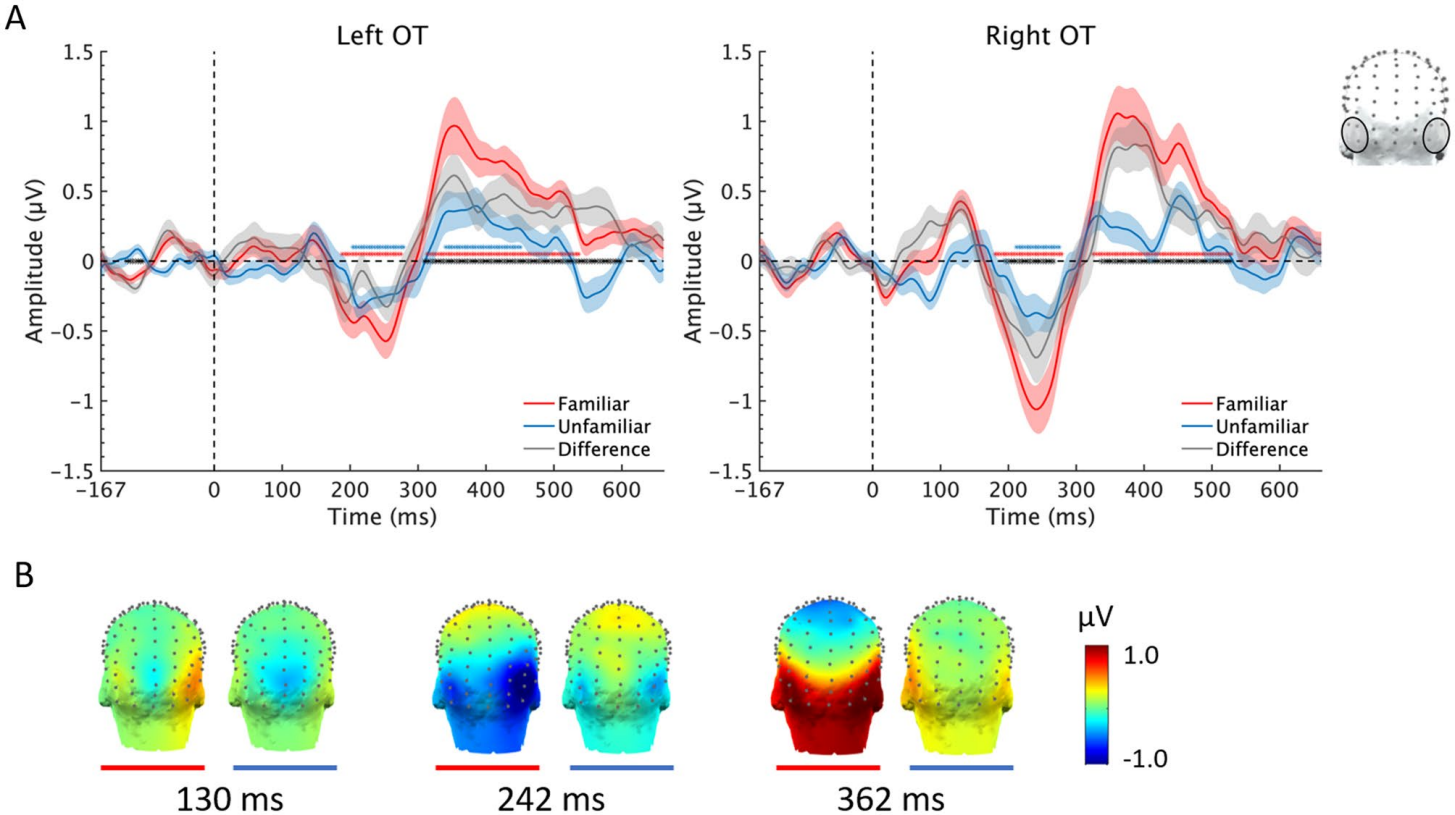
Time course of the FIR response. (**A**) Averaged waveforms over bilateral OT ROIs for the two face conditions. Shaded areas represent standard error of the mean across participants. Significant time windows are indicated with asterisks along (or above) the x-axis (red: familiar face condition, blue: unfamiliar face condition, black: difference between the two face conditions). (**B**) Three-D topographies of FIR responses in the two conditions (familiar face condition: red underline; unfamiliar face condition: blue underline) at three time-windows: 130 ms, 242 ms and 362 ms.

Here we show the temporal dynamics of FIR responses in the two face conditions and their topographies at three time-windows (Fig. 7). Compared to unfamiliar faces, the FIR response to familiar faces showed two obvious deflections located over bilateral occipito-temporal regions: a first negative waveform emerging at about 180 ms after stimulus onset and peaking at about 242 ms, and a following positive deflection appearing at about 300 ms and peaking at about 362 ms. An early positive deflection was also observed in the right OT region peaking at around 110 ms after stimulus onset. The FIR response difference between the two conditions was observed in the time window at around 158–271 ms and 337–530 ms in the right OT region, and at around 310–600 ms time window in the left OT region with a cluster-based method (see methods section).

## Discussion

Coupling FPVS with high-density EEG we report, quantify and characterize a large neural advantage in implicitly generalizing across widely different views of the same face identity when this identity is familiar as compared to when it is unfamiliar, i.e., a neural FIFE. In the present paradigm, an oddball response can only emerge in the EEG spectrum if the face identity appearing every five stimuli differs consistently from the base images of the same identity. Since these base images are highly variable (Fig. 1), perceived between-person variability of the faces must go above and beyond perceived within-person variability to generate a neural oddball response. This is the case here where, despite no difference in absolute neural response to familiar and unfamiliar faces (response at 6 Hz and harmonics; Fig. 5), the advantage in FIR manifested as a 3.44 times increase of amplitude for familiar as compared to unfamiliar faces over the most sensitive OT regions. Or, to put it differently, the FIR response to unfamiliar faces was only 29% as high as the response to familiar faces. Given that the levels of familiarity/unfamiliarity of the participants with the faces were only measured subjectively (i.e., without validation by an independent behavioral task) and were not even at maximal contrast (as expressed through responses to the post-EEG face questionnaire, see methods), this value of 3.44 is probably an underestimation of the magnitude of the neural FIFE.

The neural FIFE observed here is in line with, and complements, the numerous behavioral studies that have found an advantage at matching faces for their identity—even when they are presented concurrently—when they are familiar as compared to being unfamiliar^4–6,37^. However, as described in the introduction, the effect is difficult to quantify with explicit behavioral tasks, and usually only reported in terms of differences in accuracy rates (e.g., with no information about differences in response times to match familiar as compared to unfamiliar faces; see the above-mentioned behavioral studies). Moreover, with (usually untimed) explicit behavioral tasks, the FIFE is not only variable across individuals and stimulus sets but also across task instructions^1,38^*.* Here, despite the simple orthogonal task used, the magnitude of the FIFE was also highly variable across individuals (Fig. 4). While it may be tempting to attribute this variability to different levels of familiarity of the participants with the presented faces, there was little variance at this level, at least in terms of self-reports, except for the case of prosopdysgnosia tested. Most importantly, a large part of the variability in the amplitude of such EEG responses across individuals, even *differential* responses as measured here directly in our paradigm, is likely to reflect variability in anatomical configuration (i.e., distance of sources relative to the scalp, skull thickness, cortical folding,...) rather than in the magnitude of the neural sources^39,40^.

Here, the neural FIR response was also highly variable across identities both for familiar and unfamiliar faces (Fig. 3C). This observation has practical implications, suggesting that several face identities should be used to assess the effect (see below). These differences across stimulus sets are likely to reflect different degrees of familiarity, e.g., FID1 may be more familiar overall, but could also be due to intrinsic differences in within-person variability (e.g., different views of FID1 may be less different than different views of FID4). Given the very low rates of familiarity judgements for the unfamiliar faces, differences between the FIR ‘oddball’ response to unfamiliar faces (e.g., UID2 > UID4; Fig. 3C) could certainly be attributed to this latter factor. A limitation of the present study is that, for practical reasons, different image sets were used for familiar and unfamiliar faces, so that the neural FIFE may be underestimated (if within-person variability is higher for familiar faces) or overestimated (if within-person variability is lower for familiar faces). To counteract this limitation, we carefully matched our image sets for low-level cues while, most importantly, using very large and variable image sets for each base identity (n = 40) to minimize intrinsic differences in within-person variability between the familiar and unfamiliar face conditions. Although the alternative option of comparing the very same image sets in different groups of participants is appealing for future studies, it may be very difficult to realize practically, and also susceptible to over/underestimation of the effect due to the large interindividual variability found across individuals in the EEG FIR responses (Fig. 4). Future studies including a control condition with inverted faces could further strengthen our results, although we would still expect a small but significant FIFE for pictures of inverted faces. Yet another option is to test the very same participants before and after experimental or real-life familiarization with the face identities (e.g.,^26,41^). One strong argument against within-person variability being intrinsically lower for familiar than unfamiliar faces in the present study is that subject JG presenting with prosopdysgnosia showed no FIFE effect. Even more, his FIR response was, if anything, slightly larger for unfamiliar than familiar faces (Fig. 6).

How do our findings relate to previous studies that have attempted to measure neural indexes of the FIFE? Ewbank and Andrews^39^ reported identity repetition suppression across changes of head orientation in ventral face-selective regions (Fusiform Face Area [FFA], Occipital Face Area [OFA]) for familiar but not unfamiliar faces, in line with the behavioral FIFE. However, strikingly, to our knowledge, all other fMRI studies contrasting the repetition of different face images of the same person between familiar and unfamiliar faces failed to report any differences in cortical face-selective regions^43–49^, suggesting that this neural FIFE may be subtended by higher-order non-face-selective regions, such as the (left) anterior temporal lobe^43^, the left middle temporal cortex^44^, the medial temporal lobe^48^ or even the prefrontal cortex^43,44^.

In the same vein and while many studies have showed that individuation of faces occurs already in this early time window^47,48^ (for review, see^49^), the larger occipito-temporal N170/M170 component in EEG/MEG for different identities as compared to the repetition of the same identity is not consistently modulated by familiarity (e.g., small effects in^53,54^; no or opposite effects in^55,56^). Visual stimulus repetition effects on a later component, the N250r ('r' for repetition^54,55^ (for review, see^56^) are consistently larger for familiar than unfamiliar faces^60–63^. However, the N250r effect is smaller when different images of the same identity are repeated compared to when identical face images are repeated^58^. Thus, the larger N250r effect for familiar faces could be driven by physical similarities/differences between familiar and unfamiliar face stimuli, especially since one or only a few images of a given individual face are used. Finally, a relatively recent FPVS study built on the original paradigm of Liu-Shuang and colleagues^21^ showed a small advantage (< 10%, just above statistical threshold only in the right occipito-temporal region of interest) in the frequency-domain for familiar(ized) over unfamiliar faces^64^. However, there was only one image per face identity, and familiar faces had been seen (i.e., familiarized) in the exact same image format before the EEG testing. Moreover, as the authors acknowledged, only 16 of the 24 participants (66%) showed a larger response for familiar faces, meaning the effect of familiarity could not be identified at the level of individual participants.

Thus, to the best of our knowledge of the literature, the present findings of such a large and consistent neural FIFE, significant at the individual level within a few minutes of testing, is truly unique, and represents the first evidence of an effect in line with the large and consistent behavioral advantage for matching familiar as compared to unfamiliar faces for their identity. We attribute the large neural effect found here to several factors. First, the significant advantage of the FPVS-EEG approach in SNR. This high SNR is due to the high rate of stimulation, each participant being tested with a large number of trials (i.e., contrasts between a change of face identity and the repeated identity), i.e., 656 (8 × 83) across the 8 stimulation sequences, as well as to the concentration of the signal on narrow (frequency) bins of interest contaminated by very little noise estimated only in neighboring bins^22,65,66^. Second, while previous studies generally used two images per face identity^44,47^, with a substantial change between the two, we presented a very large set (40) of images of the same identity across changes of viewing conditions, with many intermediate views facilitating generalization (Fig. 1). Third, although the face identities were not presented simultaneously as in behavioral studies, each face identity was presented immediately after one another, facilitating both the association between different views of the same face identity and the detection of contrast with different face identities. In contrast, previous studies testing for modulation of neural responses by face identity repetition involved a delay of several hundreds of milliseconds^57,67^ to tens of seconds^44^, or compared separate blocks of the same identity to blocks of different identities^48^. Finally, in the present electrophysiological study, rather than focusing on specific—subjectively defined—time-domain components (e.g., N170/N250r) or (usually weakly powered) multivariate pattern analyses in the time-domain, we measured the whole neural FIFE in the frequency-domain, capturing all differences that occur at a 1.2 Hz rate independently of specific windows of interest. Yet, we also provide complementary time-domain analysis not restricted to specific components that shed new light on the time-course of the neural FIFE. Interestingly, our time-domain analysis on an individual selection of channels based on frequency-domain effects points to a FIR neural response in this paradigm emerging at about 180 ms following stimulus onset, with an effect prolonged until about 600 ms (Fig. 7).

Does the present study help clarify the source of the FIFE? In view of the robustness of the behavioral effect but also its absence in non-human primates^8,9^, resolving this issue may be particularly informative regarding the nature of human face recognition. Based on the robustness of the behavioral FIFE observed across different tasks, a number of authors have claimed that fundamentally different representations and processes must be at play for familiar and unfamiliar faces^7,12,16,68^. In particular, it has been suggested that contrary to familiar faces, unfamiliar faces are matched for their identity using low-level—iconic or image-based—cues^7,12^. However, this claim is unsubstantiated and incoherent with multiples sources of evidence, e.g., that discrimination of unfamiliar face identity is largely affected by manipulations that preserve differences in low-level cues between stimuli such as picture-plane inversion or contrast-reversal^69–72^, that it involves category-selective regions of the ventral occipito-temporal cortex rather than primary visual areas^40,48,73–75^, and that it is systematically affected in prosopagnosia, a high-level visual recognition disorder^11^. The findings of the present study are also in disagreement with the view that low-level cues only, or mainly, subtend the matching of unfamiliar faces for their identity: despite the wide variability of images, the much-reduced FIR response for unfamiliar faces was still significant at the group level, over the same OT regions (although with a smaller right lateralization) and same time-windows as for familiar faces (Figs. 3 and 7).

During untimed behavioral tasks requiring to associate different views of the same face identity, it is much more likely that the advantage for familiar faces is due to associations of multiples ‘codes’^37^, in particular non-visual semantic or even verbal associations (e.g., the same names evoked by two different views of the same face can be associated without even visually comparing the faces^76–79^). In the present EEG study, however, the contribution of non-visual semantic/verbal associations to match different views of the same familiar face identity must be limited, due to both the lack of explicit task and the fast presentation of stimuli. Moreover, the neural FIFE emerges relatively early, i.e., at about 180 ms following stimulus onset, making it unlikely that it is due to the online recruitment of semantic/verbal associations to link the different face views. Instead, we suggest that it is the strong association with semantic/verbal information *in the individual’s past experience* with these faces that provide the ’glue’ to link different visual views of the same face identity, leading to this robust neural FIFE^80^. While the time course, lateralization and scalp topography of the present neural FIFE suggest that these associations between different visual views of the same familiar face identity are recorded at least partly in the cortical face network of the ventral occipito-temporal cortex, future studies adapting the present paradigm to fMRI (as for face localizer experiments^81,82^) or intracranial human EEG will be necessary to resolve this issue. Based on the present observations, and contrary to previous studies as reviewed above, we predict to find larger effects of face identity repetition for familiar faces already in core face-selective regions.

Finally, let us conclude by briefly raising some practical implications of our findings. An impressive aspect of the evidence provided here is the high sensitivity of the paradigm: every individual tested, but the case of prosopdysgnosia JG, showed significant neural FIR responses to familiar faces (following 10 minutes of testing), and a numerical advantage for familiar over unfamiliar faces (i.e., a neural FIFE; Fig. 4). In all but two participants (P9 & P13) tested individually, there was a statistically significant advantage of the FIR response to familiar faces over unfamiliar faces. We think that these findings are remarkable, especially considering the difficulties encountered by previous neural (EEG/MEG and fMRI) studies to reveal a neural correlate of the behavioral FIFE at the group level, let alone in individuals, but also because the whole recording time of the present experiment is quite short (i.e., about 10 minutes for each category of faces). Moreover, while we ensured that (un)familiar faces were (un)familiar for every participant, the familiarity contrast could have even been stronger overall and strengthened by independent behavioral measures (see methods and discussion above). This high sensitivity coupled with the high specificity (i.e., no hint of a neural FIFE in the case of prosopdysgnosia JG, in line with behavioral data) and the focal localization of the effect in all individuals (i.e., allowing to use a small number of recording channels) makes this paradigm highly desirable for implicit evaluation of human face identity knowledge in individual participants, with practical implications in clinical (i.e., as biomarkers of neurodevelopmental or neurodegenerative disorders in face identity recognition, e.g., Autism Spectrum Disorder or Alzheimer’s disease^83,84^) and forensic research for instance^85^. In this vein, providing that the stimulus set remains large and is adequately adjusted to new populations, future studies could build on the present paradigm to evaluate (1) the relationship between the magnitude of the neural FIFE and the degree of familiarity with the faces (i.e., beyond a simple contrast between familiar and unfamiliar faces) and (2) its sensitivity to personally familiar and/or familiarized (learned) face identities on an individual basis.

## Methods

### Participants

Seventeen Caucasian participants participated in the experiment. The data of three participants were excluded from further analyses due to excessive noise/muscular artifacts during EEG recording. The final sample consisted of 14 participants (6 females, mean age 22.72 ± 1.96 years, all right-handed). All participants had normal or corrected-to-normal vision. None reported to have a history of neurological or psychiatric disorder. The Biomedical Ethical committee of University of Louvain (ref no. B403201111965) approved the study. Research was undertaken in accordance with relevant guidelines and regulations and all participants signed a written informed consent prior to taking part in the experiment. Prior to EEG testing, all participants were tested with the computerized Benton Facial Recognition Test (BFRT-c^86^) to assess their face discrimination abilities. They all performed within the normal range, with a mean score of 44.2/54 (± 2.5) (range: 42–50) and a mean response time (RT) per item at 8.1 ± 3.1 s.

### Case participant JG

Besides typical participants, the study also included an age-matched participant who reported severe difficulties at recognizing face identities for his entire life. Participant JG is a right-handed 20-year-old male. According to his self-description, he has always been terrible at recognizing people’s identity from their face, instead relying more on people’s voice or their clothing style. He claims to have difficulties at recognizing his own parents and even his own face on a photograph or in a mirror. An MRI of his brain showed no abnormality and JG has an intellectual efficiency in the superior range (total IQ of 124; WAIS-IV). Contrary to all other participants, he was severely impaired at the BFRT-c, with a score of 28/54 and more than 17 min to perform the whole test. He was also severely impaired at the Cambridge Face Memory Test (CFMT^87^, score at 22/72). At a two-alternative forced choice delayed matching task with pictures of faces and cars presented at upright and inverted orientations (as described in experiment 4 of^88^), he performed at chance for faces (upright: 47.2%, inverted: 58.3%), taking between 6 and 7 s by trial. In contrast, his performance with pictures of cars was excellent (97.2% and 91.7%, respectively), although a bit slow (mean RT at 2051 ms and 1941 ms; see^89^ for normal participants’ typical RTs at around 1000 ms). Directly related to the goal of the present study, JG was tested at matching simultaneously presented natural images of familiar and unfamiliar faces for their identity (see^11^ for task description). He showed no advantage at matching familiar as compared to unfamiliar faces for their identity (63.6% vs. 77.3%, respectively) and was extremely slow at that task (mean RT at 12.3 s vs. 13.3 s, respectively). In short, participant JG appears to suffer from developmental prosopagnosia^87^, or more accurately *prosopdysgnosia*^15,90^, and constitutes an ideal case to contrast with typical participants in our EEG study.

### Stimuli

Two sets of stimuli were used. For the familiar face set, 14 Caucasian male celebrities were selected according to their popularity/familiarity among the Belgian young adult population. Most of them were French or American actors or politicians. Four (Matt Damon, Jean Dujardin, Nikos Aliagas, and Leonardo Di Caprio) were used as base identities (40 natural images each, varying in lighting conditions, head orientation, facial expression, etc.), while the other 10 were used as oddball identities (1 image each). This ratio was used to ensure that the number of image repetitions was constant between base and oddball stimuli within a stimulation sequence (even though differences at this level do not affect the ‘oddball’ response with widely natural images^19^). For the unfamiliar face set, there were also 14 male celebrities, which were unfamiliar to the Belgian young adult population. Four of them (Adam Garcia, Anthony Delon, Daniel Goddart and Thierry Neuvic) were used as base identities (with 40 natural images each), and the remaining 10 were used as oddball identities (1 image each). All face identities were selected to be similar in general physical appearance (age, hair color, no distinctive facial feature such as a mole, etc.). Mean age of the celebrities in the familiar and unfamiliar face sets was 45 ± 5.16 years and 45.36 ± 4.36 years, respectively. All face images were resized to 250 × 200 pixels, extending a visual angle of approximately 9.1° in height and 8.5° in width when viewed from 80 cm away.

### Image analysis

Images of familiar and unfamiliar faces (170 images in each set) were compared in several ways to exclude potential factors that could drive FIR response differences between the two face conditions (Fig. 2). All statistical comparisons between the two face sets were carried out with non-parametric permutation tests (10,000 permutations, 2-tailed, *p* < 0.05). First, there were no global luminance and contrast differences between the two sets of stimuli (both *ps* > 0.1, Fig. 2A,B). Second, although face images varied greatly in head orientations among each other, there was no significant difference between the two sets (*p* > 0.1, Fig. 2C). The head orientation variation of each face image was quantified by measuring the distance from the tip of the nose to the left and right pupil, respectively and calculating the ratio of the two distance-metrics (i.e., left/right). A value close to 1 indicates that the face image is in frontal view. Third, the face area was defined by the boundaries between forehead (highest point of the face) and the chin (lowest point of the face), and the boundaries between left and right cheek (most left/right point of the face). The ratio of face areas between the two sets were statistically different (*p* < 0.001, Fig. 2D) but the mean difference was only about 2.5 ± 7.5% (familiar, 35.56% of the whole image area, unfamiliar, 33.03% of the whole face area). To counteract this face area difference, the size of face images during each presentation cycle was randomly changed from 80 to 120% for both conditions. Finally, we compared the difference in amplitude spectrum between the two sets of face images. Figure 2E shows the radial average of the amplitudes as a function of spatial frequency. Note that we used rectangular images in the study, so the circular average of the amplitude spectrum only took the center part of the two-D amplitude spectrum that matched the smaller dimension of the image (i.e., 200 pixels, 100 cycles/image). The energy/contrast decreased as the spatial frequency increased for both sets of stimuli with no obvious differences (left panel). This observation was further confirmed when the average amplitude spectra were converted into log–log scale and fitted with linear functions (right panel). No significant slope difference was found (*p* > 0.1). The spectral signatures shown in the contour plots also showed similar patterns between the two face sets (Fig. 2F).

### FPVS procedure

During each sequence, different face images (40 in total) of one of the base face identities were presented at a fixed rate of 6 Hz over 74 s, including 2 s of fade-in at the beginning of the sequence and 2 s of fade-out at the end. Images of different face identities were embedded every 5th image (i.e., at 6 Hz / 5 = 1.2 Hz; Fig. 1). All face stimuli were presented through square-wave modulation of contrast (with a 50% duty cycle^91^). To avoid low-level visual effects related to face identity changes, image size varied randomly between 80 and 120% in 10% steps at each stimulation cycle^21^. In each stimulation sequence, participants were presented with all familiar or all unfamiliar faces. There were four familiar face sequences and four unfamiliar face sequences, each with a different base identity selected. The eight sequences were repeated once, leading to a total of 16 stimulation sequences. The sequence order was pseudo-randomly assigned across participants. During each sequence, the participants’ task was to perform an orthogonal task by monitoring and responding to the color change of a central fixation point (from blue to red, non-periodic, 500 ms). The testing session took about 20–25 min to be completed (including breaks).

Immediately after their EEG recording, participants were asked to complete a face questionnaire to assess their knowledge of the faces used in the experiment^23–25^. In the questionnaire, each of the 28 face identities (14 familiar and 14 unfamiliar) was shown and the participants had to respond whether the faces were familiar to them (yes, no, uncertain). If the face was familiar, the participants were also required to rate its familiarity on a 10-point Likert scale (from “rarely seen in the media” to “very frequently seen in the media”) and report its name and occupation. On average, participants were familiar with 10.3 (± 3.1) out of the 14 familiar faces (73.5% ± 22.5), and 0.4 (± 1.2) out of the 14 unfamiliar faces (3.1% ± 8.3). The mean familiarity ratings to the four familiar base identities were 8.50 ± 1.45 for ID1, 7.71 ± 2.55 for ID2, 5.93 ± 3.17 for ID3, and 7.43 ± 1.95 for ID4. The mean rating to the four unfamiliar base identities were at 0 ± 0 for ID1, 0.43 ± 1.6 for ID2, 0.29 ± 0.82 for ID3 and 0.21 ± 0.80 for ID4. Importantly, none of the unfamiliar faces that were rated as known were identified by their name.

### Behavioral analysis and results

We calculated the RTs for the participants to respond to the orthogonal color detection task relative to the onset of the color change. As in previous studies (e.g.,^23,25^), responses were considered correct if they occurred between 150 to 1000 ms following the onset of the color change. Behavioral data of one participant was lost due to technical issues. With the remaining 13 participants, the mean accuracy across individuals and conditions was close to ceiling (97.14% ± 2.72%), and the mean RT was about 505 ms (± 51 ms).

### EEG recording

Similarly to what has been described in previous studies (e.g.,^23,25^), participants were tested in a quiet and low-lit lab room and their brain activity was recorded with a high-density 128-channel ActiveTwo Biosemi EEG system (Biosemi, Amsterdam, The Netherlands) at a 512 Hz sampling rate. The magnitude of the offset of all electrodes, referenced to the common mode sense (CMS), was held below 30 µV. Vertical and horizontal electrooculogram (EOG) was recorded using four additional flat-type active-electrodes: two electrodes above and below the participant’s right orbit and two lateral to the external canthi of the two eyes. The visual stimuli were displayed in the center of the screen on an LED monitor (BenQ XL2420T) with 1920 × 1080 screen resolution and a monitor refresh rate of 120 Hz.

### EEG analysis

EEG data was preprocessed in the open-source software Letswave5 (https://github.com/NOCIONS/Letswave5), running in MATLAB R2016a (MathWorks, USA) according to well-validated procedures as reported previously^24,25^. After importation, continuous blocks of recording were aligned to the first block to correct for vertical jumps due to voltage drift during pauses. EEG data were first band-pass filtered between 0.05 to 100 Hz with a 4th order zero-phase Butterworth filter and then notch-filtered to remove the 50 Hz frequency and 2 following harmonics (100 and 150 Hz, width 0.5 Hz). Data were downsampled to 256 Hz to reduce computational load. The whole EEG trace was then segmented relative to the starting trigger of each sequence, with an additional 2 s before and after each sequence (−2 to 76 s). Eyeblink artifacts more than 0.2 times/s on average were corrected by applying independent component analysis (ICA) on one participant (P10)^19^. No interpolation of bad channels was required in our participants. Finally, the EEG sequences were re-referenced to the average of all 128 electrodes.

### Frequency-domain analysis

The methodology for carrying out frequency-domain analysis was the same as described in previous FPVS studies (e.g.,^23,25^) and started by further segmenting the EEG epochs to reduce the sequences to an integer number of 1.2 Hz cycles^19^. During this process, the first and last 2 s of each presentation sequence, corresponding to fade-in and fade-out periods, were discarded to remove eye movements and muscle artifacts related to the onset and offset of the flickering stimuli. The resulting cropped epochs were 69.18 s long, with 83 ‘oddball’ face presentation cycles. A Fast Fourier Transform (FFT) was applied on these cropped epochs to extract amplitude spectra for each participant, with a frequency resolution of 0.0145 Hz (1/69.18 s). Two baseline correction transforms were then computed using the 48 neighbouring bins surrounding the frequency bins of interest (24 bins on each side, excluding the immediately adjacent bins in case of remaining spectral leakage): (1) the frequency bins of interest were divided by the EEG noise to estimate their signal-to-noise ratio (SNR), allowing to better visualize small responses (Fig. 3A) and (2) EEG noise in the neighbouring bins was subtracted from the frequency bins of interest (baseline subtraction, SBL) to quantify responses in microvolts (µV) across harmonics.

Consistently with previous studies^23–25^, responses at 1.2 Hz and 6 Hz were accompanied by multiple harmonics. To select harmonics to consider for further analysis, we computed the grand-average on EEG responses across all participants, all channels and both conditions and applied a z-score transform on these grand-averaged data. Consecutive harmonics that were significant at a threshold of z > 2.3 (*p* < 0.01, one-tailed, signal > noise) were then selected to compute the sum of harmonics^23–25^. Following this procedure, the first 5 harmonics of the FIR frequency (i.e., 1.2 Hz, 2.4 Hz, 3.6 Hz, 4.8 Hz, and 7.2 Hz, excluding the 6 Hz), and the first 10 harmonics of the general face frequency (i.e., 6 Hz, 12 Hz, and up to 60 Hz) were selected and summed to quantify the FIR and base response, respectively. EEG responses were statistically tested at two levels: (1) across the whole scalp (all 128 channels) and (2) at local regions-of-interest (ROIs). These ROIs were defined based on grand-averaged responses at the frequencies of interest (1.2 Hz and 6 Hz, along with their harmonics) across the two conditions and consistently with previous studies that used the same frequency-tagging approach to measure familiar face identity recognition^23–25^ and unfamiliar face individuation^21,92–94^: 10 posterior channels (P7&8, P9&10, PO7&8, PO9&10, PO11&12) were included in a bilateral occipito-temporal (OT) ROI, and 12 middle posterior channels (POOz, Oz, OIz, Iz, POO5&POO6, O1&2, POI1&2, I1&2) were included in a middle occipital ROI. The bilateral OT ROI was also divided into a left OT ROI (P7, P9, PO7, PO9, PO11) and a right OT ROI (P8, P10, PO8, PO10, PO12) to investigate hemispheric differences in the FIR responses.

To measure the FIR response at the individual level, we calculated the z-score based on the summed-harmonic response at 1.2 Hz (including the first 5 harmonics) and their 48 neighbouring bins over both left and right OT ROIs. A response above a z-score threshold of 1.64 (*p* < 0.05, one-tailed, signal > noise) was considered as significant, indicating that the FIR response was significantly larger than the neighbouring EEG noise.

### Time-domain analysis

Time-domain analyses of the EEG data were carried out following a similar procedure as used in previous studies using the same frequency-tagging approach^19,25,95^. Re-referenced EEG signal was low-pass filtered with a 30 Hz cut-off (4th order Butterworth filter), and cropped into an integer number of cycles of 1.2 Hz (from 2 to 71.18 s, 83 face presentation cycles). After that, the general face presentation frequency and its first 5 harmonics (up to 30 Hz) was removed with narrow band notch-filtering (4th order, width = 0.05). The EEG waveforms were then segmented into smaller epochs containing five stimulation cycles (i.e., 833 ms), including four base face images (FID1) and one identity change face image (FID2) with a pattern of ‘FID1-FID2-FID1-FID1-FID1’. Epochs were averaged and baseline-corrected relative to the first base face stimulus presentation (−167 to 0 ms). This analysis was performed in individual participants before averaging at the group level.

To determine the time-windows that showed a significant FIR response difference between the familiar and unfamiliar face conditions, we ran a cluster-based non-parametric permutation t-test (10,000 permutations, with a threshold of *p* < 0.05, two-tailed) on the post-stimulus onset time points (0–667 ms)^96,97^. The null hypothesis was that there was no difference between familiar and unfamiliar face conditions at any time point. For each permutation, we compared the waveform difference between two conditions with the condition labels randomly shuffled across participants using a paired t-test and calculated the cluster-level statistics as the sum of t-values in the consecutive time points with p-values less than 0.05^98^. We then took the maximum cluster-level statistic for each permutation to make a non-parametric reference distribution of cluster-level statistics. We rejected the null hypothesis if the cluster-level statistics for any consecutive sequence in the original data was larger than 97.5% or smaller than 2.5% of the values in the null distribution. We also applied the same cluster-level analysis to find significant time windows against zero for both the familiar and unfamiliar face conditions.

## Data Availability

The scalp EEG datasets analyzed in the current study are available in an OSF repository, https://osf.io/te4sh/?view_only=e04ff7c9981d41a890d7d751fdbb8f89.
